# Compensatory Neural Recruitment for Error-Related Cerebral Activity in Patients with Moderate-To-Severe Obstructive Sleep Apnea

**DOI:** 10.3390/jcm8071077

**Published:** 2019-07-22

**Authors:** Ping-Song Chou, Sharon Chia-Ju Chen, Chung-Yao Hsu, Li-Min Liou, Meng-Ni Wu, Ching-Kuan Liu, Chiou-Lian Lai

**Affiliations:** 1 Department of Neurology, Kaohsiung Medical University Hospital, Kaohsiung Medical University, Kaohsiung 807, Taiwan; 2 Department of Neurology, Faculty of Medicine, College of Medicine, Kaohsiung Medical University, Kaohsiung 807, Taiwan; 3 Department of Medical Imaging and Radiological Sciences, Kaohsiung Medical University, Kaohsiung 807, Taiwan; 4 Department of Medical Research, Kaohsiung Medical University Hospital, Kaohsiung 807, Taiwan

**Keywords:** action monitoring, error positivity, error-related negativity, functional magnetic resonance imaging, modified Flanker task, obstructive sleep apnea

## Abstract

(1) Background: Although it is known that obstructive sleep apnea (OSA) impairs action-monitoring function, there is only limited information regarding the associated cerebral substrate underlying this phenomenon. (2) Methods: The modified Flanker task, error-related event-related potentials (ERPs), namely, error-related negativity (ERN) and error positivity (Pe), and functional magnetic resonance imaging (fMRI) were used to evaluate neural activities and the functional connectivity underlying action-monitoring dysfunction in patients with different severities of OSA. (3) Results: A total of 14 control (*Cont*) subjects, 17 patients with moderate OSA (*m*OSA), and 10 patients with severe OSA (*s*OSA) were enrolled. A significant decline in posterror correction rate was observed in the modified Flanker task when patients with *m*OSA were compared with *Cont* subjects. Comparison between patients with *m*OSA and *s*OSA did not reveal any significant difference. In the analysis of ERPs, ERN and Pe exhibited declined amplitudes in patients with *m*OSA compared with *Cont* subjects, which were found to increase in patients with *s*OSA. Results of fMRI revealed a decreased correlation in multiple anterior cingulate cortex functional-connected areas in patients with *m*OSA compared with *Cont* subjects. However, these areas appeared to be reconnected in patients with *s*OSA. (4) Conclusions: The behavioral, neurophysiological, and functional image findings obtained in this study suggest that *m*OSA leads to action-monitoring dysfunction; however, compensatory neural recruitment might have contributed to the maintenance of the action-monitoring function in patients with *s*OSA.

## 1. Introduction

Obstructive sleep apnea (OSA) is one of the most common forms of sleep-disordered breathing, which is characterized by repetitive narrowing and opening of the upper airway during sleep, resulting in a cyclical breathing pattern accompanied by hypoxia and reoxygenation (intermittent hypoxia), and brief awakenings from sleep (sleep fragmentation) [1]. Intermittent hypoxia and sleep fragmentation can induce chemical and structural neuronal injury, subsequently leading to prefrontal cortex dysfunction [2,3,4]. As such, OSA has been reported to affect multiple domains of frontal cognitive function, especially attention, vigilance, and executive function [5].

Action-monitoring function, an important domain of frontal cognitive function, plays a major role in adaptive behavior for early response-conflict monitoring, error detection, and then late behavioral adjustments. Therefore, action-monitoring function is important for humans to recognize errors, adjust behaviors, and optimize subsequent performances [6,7]. Action-monitoring behaviors are mediated by the anterior cingulate cortex (ACC) in the medial frontal lobe [8]. Using the modified Flanker task, action-monitoring dysfunction has been reported in patients with OSA [9]. Despite our previous reports suggesting behavioral action-monitoring dysfunction in patients with OSA, relatively less is known about the associated cerebral substrate underlying this dysfunction in people with different severities of OSA. An additional investigation using both neuroelectrophysiological response and functional connectivity is necessary on order to classify the pathological progression of action-monitoring dysfunction in patients with OSA.

Electrophysiological study is an informative and dynamic method for evaluating the neural activities underlying behaviors. Error-related negativity (ERN) and error positivity (Pe), two components of event-related potential (ERP) proposed by Falkenstein et al., are believed to reflect action-monitoring function and error processing [10]. ERN, a negative electroencephalogram (EEG) deflection that is the largest over the frontal and central midline electrodes and generally peaks at 100 milliseconds (ms) after errors, is believed to be generated in the caudal ACC [11]. Functionally, ERN is reportedly an early process of response-conflict monitoring [12,13,14]. Following ERN, a positive deflection, Pe, generated from the rostral ACC and the parietal cortex, is observed with a centroparietal maximum at approximately 300–350 ms after errors [11]. Pe is believed to reflect an independent process distinct from ERN, and it is associated with conscious error processing and late remedial action [15,16,17]. Experimentally extended wakefulness and sleep deprivation can result in decreased amplitudes of ERN and Pe, indicating impaired action-monitoring function [18,19,20,21].

Although electrophysiological study offers excellent temporal resolution, the spatial resolution achieved using the technique is not satisfactory. Functional magnetic resonance imaging (fMRI) is another practical tool with excellent spatial resolution that can be used to evaluate changes in the cerebral response and the functional connectivity underlying behaviors. Studies using fMRI and cognitive tasks pertaining to attention, mismatch, and response inhibition have shown that patients with OSA exhibited altered cerebral activation in the frontal region and the ACC [22,23,24]. Among fMRI techniques, resting-state fMRI provides a convenient and fundamental assessment tool to measure brain activity [25].

However, to the best of our knowledge, no study has yet investigated the differences in neural activities and functional connectivity underlying action-monitoring dysfunction in patients with different severities of OSA using electrophysiology (i.e., ERN and Pe) and neuroimaging (i.e., fMRI). Considering that patients with OSA exhibit behavioral action-monitoring dysfunction, we hypothesized that patients with different severities of OSA would exhibit different patterns of neural activities and functional connectivity underlying action-monitoring dysfunction. We tested our hypothesis using the modified Flanker task to evaluate the action-monitoring function and to obtain electrophysiological measurements, namely, ERN and Pe, and fMRI, for examining neural activities and the functional connectivity in cerebral regions related to action-monitoring in patients with OSA of different severities.

## 2. Materials and Methods

### 2.1. Participants

A cross-sectional case–control design was adopted in this study. A total of 27 patients with moderate-to-severe OSA (apnea–hypopnea index (AHI) ≥ 15 counts/h) were recruited from Kaohsiung Medical University Hospital in Southern Taiwan, and 14 healthy controls were recruited from the general community between 2012 and 2015. All study participants were categorized into the following three groups based on their AHI values: Control (*Cont*) participants with AHI < 5 counts/h, participants with moderate OSA (*m*OSA) with 15 counts/h ≤ AHI ≤ 30 counts/h, and participants with severe OSA (*s*OSA) with AHI > 30 counts/h. The AHI ranges of the three groups were defined according to the American Academy of Sleep Medicine [26].

After including the participants in the study, each participant received a comprehensive medical evaluation, including cognitive assessment conducted using the Chinese version of the Cognitive Abilities Screening Instrument (CASI) [27], and a complete overnight polysomnography (PSG) examination. All participants were qualified with CASI cognitive evaluation for normality with a total score of at least above 85. To avoid inclusion of participants with potential OSA in the *Cont* group, all *Cont* individuals with AHI ≥ 5 counts/h were excluded. When participants had completed the overnight PSG examination, they underwent the modified Flanker task to evaluate their action-monitoring function, and meanwhile, ERPs were acquired using EEG. Then, a resting-state fMRI was performed for each participant with instruction to lie in the MRI scanner without any specific thoughts and to be awake.

We excluded participants with a history, or clinical evidence, of neurological or psychiatric disease, those with major organ failure, those receiving medical therapy that might affect their cognitive function (e.g., a prescription of benzodiazepines, anticholinergics, antipsychotics, or other potential sedatives), and those with CASI scores lower than the cutoff value (adjusted for age and level of education) [28]. In addition, all participants were right-handed users and claimed that they have not had any medical experience due to of sleep-disordered breathing. None of them consumed alcohol, caffeine, nicotine, or any cognition-enhancing medications on the day of testing. This study was conducted in accordance with the Declaration of Helsinki and approved by the Institutional Review Board of Kaohsiung Medical University Hospital (KMUHIRB-2012-03-09(II)). Participation was voluntary, and all participants provided written informed consent.

### 2.2. Modified Flanker Task and Assessments of Error-Related ERPs

The modified Flanker task was performed to elicit ERPs in this study. Briefly, five visual stimuli were presented in the middle of a computer monitor, and participants were instructed to indicate the direction of a central target stimulus. In congruent trials, the nontarget stimuli corresponded to the same direction as the target (<<<<< or >>>>>), whereas in incongruent trials, the nontarget stimuli corresponded to the direction opposite to that of the target (<<><< or >><>>). Finally, visual feedback was provided to all participants based on the accuracy of their responses. The formal task involved five sessions, each comprising 100 trials. The order of stimuli randomly differed in each session.

During the task, the participants responded to congruent and incongruent trials, which were presented randomly in a trial ratio of 1:4. Meanwhile, electrophysiological signals were recorded by continuous EEG, which was performed using Ag/AgCl electrodes placed on six locations of the central scalp (Fz, FCz, Cz, CPz, Pz, and Oz) based on the international 10–20 system montage; all electrodes were referenced to linked earlobes. Electrode impedance was maintained at lower than 5 kΩ. The EEG signals were filtered with a 0.01–40 Hz bandpass filter using a SynAmps amplifier (NeuroScan Inc., Charlotte, NC, USA). Continuous EEG recordings acquired at a sampling rate of 256 Hz were stored for further offline analysis.

ERPs obtained under the congruent and incongruent conditions were extracted from the continuous EEG signals and averaged separately. Response-locked ERPs, namely, ERN and Pe, were detected when participants responded incorrectly to the trials corresponding to the congruent and incongruent conditions. When a participant responded incorrectly, the ERN response was detected as the first negative peak, following errors in the time window of 50–200 ms, and the Pe response was observed as the positive component following ERN, with a latency of 200–700 ms after the error event.

### 2.3. Polysomnography

A standard overnight full-channel PSG using two validated machines (Nicolet Ultrasom, Madison, WI, USA and Respironics Alice 5, Philips, Eindhoven, Netherlands) was applied to every participant. Physiological parameters, including pulse oximetry, an EEG, an electrooculogram, an electromyogram, an electrocardiogram, nasal and oral air flow measurements, and thoracic and abdominal respiratory inductive plethysmography, were recorded. Sleep stages and sleep-related respiratory events were scored based on the criteria of the American Academy of Sleep Medicine [26]. Respiratory parameters, including the AHI, oxygen desaturation index, arousal index, percentage of time with blood oxygen saturation (SpO_2_) < 90% (SpO_2_ value < 90%), and the lowest SpO_2_, were recorded. Hypopnea was defined as a drop in peak signal excursions by ≥30% of the pre-event baseline when nasal pressure is applied for ≥10 seconds with ≥4% oxygen desaturation or a drop in peak signal excursions by ≥50% for ≥10 seconds with ≥3% oxygen desaturation or associated arousal.

### 2.4. fMRI Scan Protocol

A 6 min fMRI scan was performed to acquire the functional response of the brain in the resting state. Functional imaging covering the entire brain was acquired using a 3-Tesla GE scanner (Signa HDx, GE, Milwaukee, WI, United States) with an echo planar imaging (EPI)-based blood oxygen level-dependent sequence in the axial plane with the following parameters: Repetition time (2000 ms), echo time (35 ms), flip angle (80°), field of view (220 × 220 mm^2^), acquisition matrix (64 × 64), slice thickness (3.4 mm), slice number (28 slices/volume), and volume number (180 time points). As neuron firing is induced by endogenous or exogenous stimuli, the intensity of fMRI signals corresponds to the induced magnetic variation due to the flow of deoxyhemoglobin in blood. Furthermore, high-resolution T1-weighted images were collected along the anterior commissure–posterior commissure line using a three-dimensional spoiled gradient recalled sequence (3D-SPGR) with the following parameters: Repetition time (2.5 ms), echo time (4.38 ms), flip angle (8°), field of view (240 × 240 mm^2^), acquisition matrix (256 × 256), slice thickness (1 mm), and slice number (124 slices/volume). This T1 imaging was used as the anatomical template underlaid by functional imaging.

All participants were instructed to lie still with eyes closed, think nonspecific thoughts, and not fall asleep in the scanner. Earplugs were used to isolate the participants from the operating noise of the fMRI scanner. After the scan, the participants reported that they had not fallen asleep during scanning.

### 2.5. fMRI Data Analysis

All functional data were preprocessed using the Data Processing and Analysis of Brain Imaging (DPABI, http://rfmri.org/DPABI) suite in the MATLAB environment [29]. The first 10 volumes of imaging data were discarded for maintaining the stability of the signals. The preprocessing steps included slice timing adjustment to the middle slice between the slices of a volume, registration for motion correction between the volumes of a scanning session to the first volume, and coregistration and normalization to a standardized Montreal Neurological Institute (MNI) template averaged by 512 individuals, followed by registration to the participant’s T1-weighted anatomical image using exponentiated Lie algebra. Thereafter, the normalized data were spatially smoothed using a 4 mm isotropic Gaussian kernel, temporally filtered with a high-pass filter of 128 s, and resampled at a resolution of 3 × 3 × 3 mm^3^. After preprocessing, a linear detrending was applied to remove systematic drifting artifact before a bandpass filter was used to reserve imaging data in the spontaneous resting frequency (0.01–0.1 Hz).

Afterward, feature extraction based on the calculation of the connection intensity of the ACC functional network was performed on the preprocessed imaging data. Predefined ACC seeds were coordinated at (±7, 19, 30) in the MNI domain, according to an event-related fMRI study that conducted a modified Flanker task [30]. The reference time course of the seed was defined as the averaged time course of a spherical 8 mm region around the seed. Thereafter, the functional connectivity over the brain was calculated between the reference time course and the remaining voxels in the brain, and the connectivity was presented as the correlation intensity based on the values of the Pearson correlation coefficients. After the calculation of the correlation, an overall functional map of connection intensity was generated after thresholding the correlation intensity whose value was above statistical significance (*p* < 0.05) on an averaged map of two ACC-seeded networks.

In addition to presenting individual stationary functional network, we analyzed the interregional connectivity between regions of interest, which were extracted from the examination of the functional variation over groups. Here, we selected three action-monitoring-related regions [31,32,33], namely, the ACC, the supplementary motor area (SMA), and the precuneus, for effective connectivity analysis. Interregional interaction of the regions of interest, that is, determination of effective connectivity considering the interactive influence bidirectionally, was performed between the target regions [34]. This method proposed by the Granger causal model considers that the relationship of responsive time course between two regions (or two voxels) is bidirectionally influenced, that is, *x* → *y* and *y* → *x*. For example, the current signal (at time point *t*) at region *x* is composed of self-contribution and the contributions of the neighboring region, such as region *y*. The self-influence originates from the temporal dependence of the last signal of region *x* at time point (*t* − ∆*t*). The influence of the neighboring region originates from the functional dependence between two regions with high function correlation; that is, these regions might proceed with the same activity in response to a specific external stimulus at time point *t*. Finally, the ratio of the contributions of the neighboring region to that of self-contribution was used to represent the directional influence from region *y* to region *x* (*y* → *x*). The directional influence from region *x* to region *y* (*x* → *y*) was calculated using the same scheme used for processing thoughts.

### 2.6. Statistical Analysis

Statistical analysis was performed using SPSS (IBM SPSS 19.0, Armonk, NY, USA). Analysis of variance (ANOVA), followed by homogeneity of variance between groups (using Levene’s test), was used for continuous variables, and the chi-squared test was used to analyze categorical variables to assess the differences among the three groups in terms of demographic data, PSG parameters, behavioral performance, and error-related ERP parameters. Normality of each sample data were also examined by the Kolmogorov–Smirnov test. These checks of data quality within and between data according to items ensured the statistical validity in subsequent statistical examinations. All statistical tests were two-tailed, and an alpha value of 0.05 indicated statistical significance. In all ANOVA comparisons, we additionally considered the clinical impact with effect size (ES) given as partial eta square (*η_p_^2^*), which was defined as the sum of squares effect (*SS*_between_) over the sum of squares effect (*SS*_between_) plus the sum of squares error (*SS*_error_), that is, $\eta_{p}^{2}=SS_{between}/\left(SS_{between}+SS_{error}\right)$ [35]. ES is less limited by the sample size rather than by the *p* value significance tests, and it is appropriate to explain the magnitude of the relationship in the population. The value corresponded to small, medium, and large ESs, respectively, as follows: 0.01 ≤ *η_p_*^2^ < 0.06, 0.06 ≤ *η_p_*^2^ < 0.14, and *η_p_*^2^ ≥ 0.14 [35,36].

In the comparison of functional connectivity among the groups, we applied two-sample *t*-tests following the extent spatial constraint for activation cluster (with alpha simulation) to examine the difference between each two groups, for example, *m*OSA vs. *Cont*, *s*OSA vs. *Cont*, and *s*OSA vs. *m*OSA, followed by assessment of normality using the Kolmogorov–Smirnov test. The resulting statistical parametric maps (*t* value) helped us observe the functional contrast between two different levels of OSA severity. Later, by conjoining these contrast maps, we were able to perform further regional analysis across the three groups. Three regions related to action-monitoring function were selected, including the ACC, the SMA, and the precuneus. The functional difference across the three groups was analyzed by one-factor ANOVA, and a *p* value of 0.05 was considered for statistical significance.

## 3. Results

A total of 10 patients with *s*OSA, 17 patients with *m*OSA, and 14 healthy adults as *Cont* were found to be eligible for participation in this study. The demographic data and the PSG parameters are presented in Table 1. Results of the chi-squared test and one-way ANOVA revealed no significant differences among the three groups in terms of gender, age, education level, and baseline CASI score, indicating no bias of sample selection in demographic data among the groups. This also reflected that the basic performance of cognitive function has no prominent difference between groups. Regarding the clinical characteristics of OSA, patients with *s*OSA exhibited significantly higher AHI, arousal index, and percentage of time with SpO_2_ (value < 90%) than the other two groups (*p* < 0.001). Patients with *m*OSA and those with *s*OSA scored higher on the Epworth Sleepiness Scale than the healthy adults (*p* = 0.036). This implied that participants had significant clinical characteristics of OSA among the groups.

### 3.1. Behavioral Response and Error-Related ERP Analysis in the Modified Flanker Task

Regarding behavioral performance in the modified Flanker task (shown in Table 1), there was a trend of increased error response rate along with the increase in OSA severity in the congruent (*Cont*: 1.6% ± 0.9%, *m*OSA: 13.9% ± 6.3%, *s*OSA: 18.7% ± 8.3%, *p* = 0.122, *η_p_*^2^ = 0.105) and the incongruent (*Cont*: 4.9% ± 1.0%, *m*OSA: 21.7% ± 7.7%, *s*OSA: 25.2% ± 8.8%, *p* = 0.091, *η_p_*^2^ = 0.118) trials, with the rates in both trials being below the level of statistical significance. Otherwise, in terms of posterror correction, based on the rate of correct response after error event, a trend of decreased posterror correction rate was observed between the three groups in the congruent trials (*Cont*: 86.3% ± 11.8%, *m*OSA: 70.4% ± 9.1%, *s*OSA: 53.2% ± 13.4%, *p* = 0.195, *η_p_*^2^ = 0.110), which was below the level of statistical significance, whereas there was a significant difference between groups in the incongruent trials (*Cont*: 89.8% ± 6.6%, *m*OSA: 74.2% ± 6.0%, *s*OSA: 64.1% ± 7.9%, *p* = 0.048, *η_p_*^2^ = 0.148). Results of the post hoc analysis revealed no significant difference between *m*OSA and *s*OSA groups in the error response rate and the posterror correction rate either in the congruent trials or in the incongruent trials.

**Figure 1:**
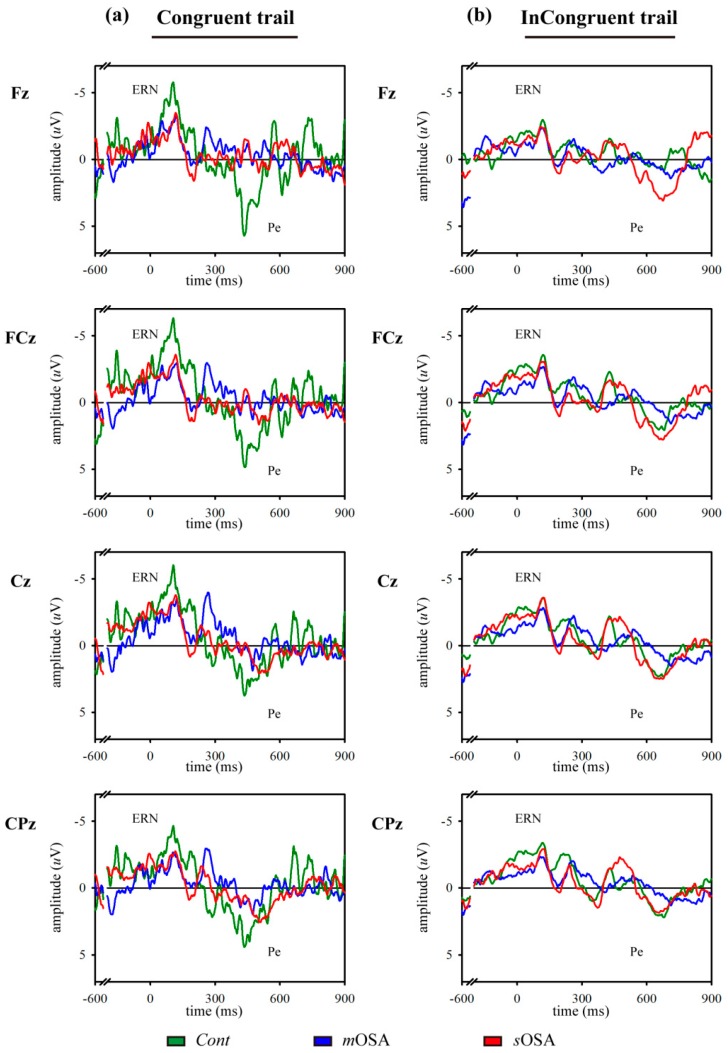
Signals of event-related potentials to error response in congruent (**a**) and incongruent (**b**) trials. Two tasking trials and four channels are demonstrated in the three groups. Results of the *Cont*, *m*OSA, and *s*OSA groups are presented in green, blue, and red, respectively. The amplitudes of ERN and Pe decreased in *m*OSA compared with *Cont*. In *s*OSA, the amplitudes of ERN and Pe increased compared with *m*OSA. A compensatory neural recruitment for action-monitoring dysfunction during the transition from *m*OSA to *s*OSA is observed. Abbreviations: *Cont*, Control group; *m*OSA, moderate OSA group; *s*OSA, severe OSA group; OSA, obstructive sleep apnea; ERN, error-related negativity; PE, error positivity.

In the analysis of neuroelectrophysiological responses, we averaged the ERP course for each specific trial condition (congruent trials or incongruent trials). The amplitudes of ERN and Pe in patients with *m*OSA, as hypothesized, were declined in the congruent trials compared with those in *Cont* subjects, but these declined amplitudes were increased in patients with *s*OSA, although the between-group differences were not statistically significant (ANOVA test, *p* > 0.05). Regarding the responses in the incongruent trials, the amplitudes of ERN and Pe exhibited similar tendencies across the study groups. Figure 1 depicts the averaged ERP course of each group in the individual channel for each tasking condition.

In summary, the modified Flanker task suggested action-monitoring dysfunction in patients with *m*OSA compared with *Cont* subjects; however, the finding of increased amplitudes of ERN and Pe in patients with sOSA might suggest a compensatory neural recruitment to maintain the action-monitoring function in these patients. The presence of a possible compensatory neural recruitment will be examined using fMRI in the next section.

### 3.2. fMRI Response and Functional Connectivity

**Figure 2:**
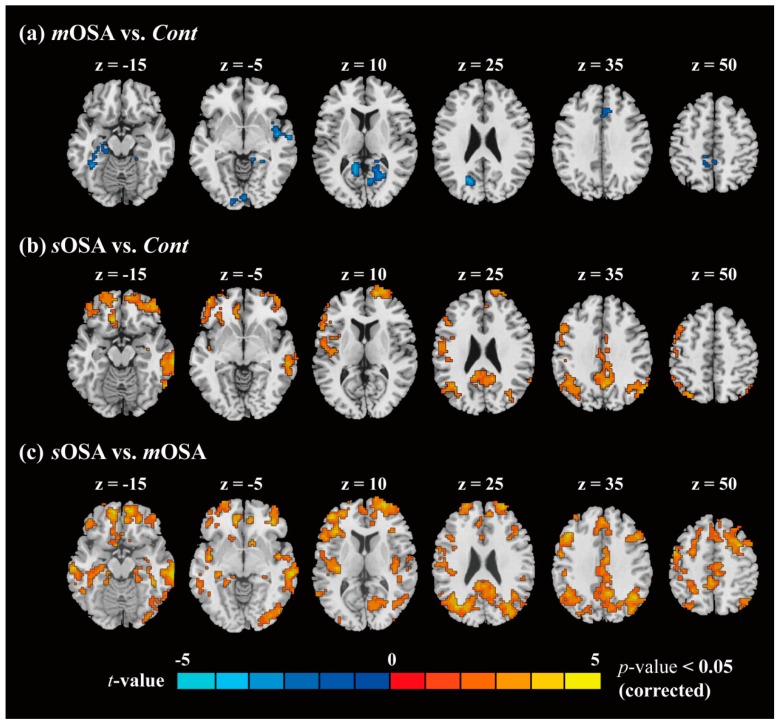
Differences in functional connection between pairs of two groups. Two-sample *t*-tests were performed on the functional connectivity data to assess the cognitive influence between all pairs of two groups as follows: (**a**) *m*OSA vs. *Cont*; (**b**) *s*OSA vs. *Cont*; (**c**) *s*OSA vs. *m*OSA. The contrast activations are below the level of statistical significance (*p* < 0.05). Abbreviations: *Cont*, Control group; *m*OSA, moderate OSA group; *s*OSA, severe OSA group; OSA, obstructive sleep apnea.

Figure 2 shows a functional comparison of the results of two-sample *t*-tests following the extent of spatial constraint for activation cluster (with alpha simulation) between pairs of each of two groups, namely, *m*OSA vs. *Cont*, *s*OSA vs. *Cont*, and *s*OSA vs. *m*OSA. The activated voxels were singled out beneath the statistical level of *p* value 0.05 plus an additional cluster constraint (*p* < 0.05). As shown in Figure 2a, patients with *m*OSA had significantly lower activations than the *Cont* group, with negative activations (*m*OSA vs. *Cont*) in the following regions: Bilateral calcarine, bilateral middle cingulate cortex, bilateral precuneus, left cuneus, left superior occipital cortex, left superior parietal cortex, right insula, right lingual cortex, and right superior temporal cortex. Figure 2b shows that patients with *s*OSA had significantly higher activations than *Cont* subjects, with positive activations (*s*OSA vs. *Cont*) in the following regions: Bilateral angular cortex, bilateral ACC, bilateral middle cingulate cortex, bilateral posterior cingulate cortex, bilateral medial frontal cortex, bilateral middle occipital cortex, bilateral precuneus, bilateral middle temporal cortex, left inferior frontal cortex, left superior-inferior parietal cortex, left precental cortex, left postcentral cortex, and right inferior temporal cortex. Altogether, a large functional variation was expected between patients with *s*OSA and those with *m*OSA (*s*OSA vs. *m*OSA, as shown in Figure 2c) in the nine brain regions. This indicates that a functional conversion might occur during the pathological progression of OSA.

Quantitative information about the activation clusters is presented in Table S1 showing the cluster size (in voxels), the maximum *t* value (*t*_max_) in the cluster (corrected), and the coordination of *t*_max_. After conjunctive comparison over the abovementioned activation maps, we selected three regions of interest over nine, namely, the ACC, the SMA, and the precuneus, which were highly associated with the pathological severity of OSA and the action-monitoring function [31,32,33], to further compare the connection within regions across the three study groups.

**Figure 3:**
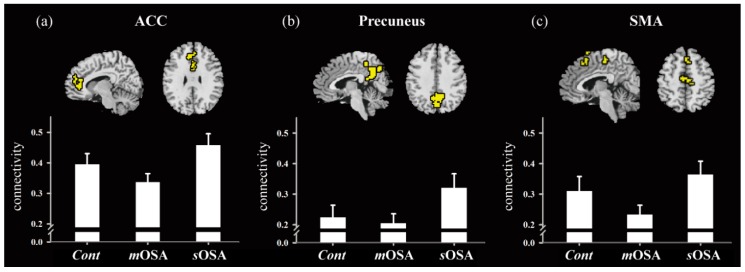
Functional comparison across groups for regions of the ACC (**a**), the precuneus (**b**), and the SMA (**c**). The activated regions shown in the figure were selected based on functional differences between the *m*OSA and *s*OSA groups. One-way ANOVA was performed, and the *p* values in each full model were 0.003, 0.004, and 0.014 for the ACC, the precuneus, and the SMA, respectively. Abbreviations: *Cont*, Control group; *m*OSA, moderate OSA group; *s*OSA, severe OSA group; OSA, obstructive sleep apnea; ACC, anterior cingulate cortex; SMA, supplementary motor area.

Figure 3 shows the functional comparisons of the ACC, the precuneus, and the SMA across the three groups along with the correlation intensity of the ACC network. A consistent trend was observed across the three groups in the form of an opened-up parabola (U shape); that is, patients with *m*OSA had the lowest functional response, whereas the functional response was increased in patients with *s*OSA. In addition, intergroup differences were evaluated by one-way ANOVA, and homogenous testing of variance was conducted for each region using Levene’s statistics. Between-group and within-group degrees of freedom were two and 34, respectively. All three regions passed the variance testing (*p* > 0.05), which indicated that they exhibited similar levels of within-group variance. Furthermore, the between-group comparison revealed significant differences, with the *p* values being 0.003, 0.004, and 0.014 for the ACC, the precuneus, and the SMA, respectively. To summarize, the functional response was deteriorated in patients with *m*OSA but significantly reactivated in patients with *s*OSA. Whether the superior functional performance in the *s*OSA group compared to that in the *m*OSA group is caused by the compensatory recruitment effect of joined neurons requires further validation.

**Figure 4:**
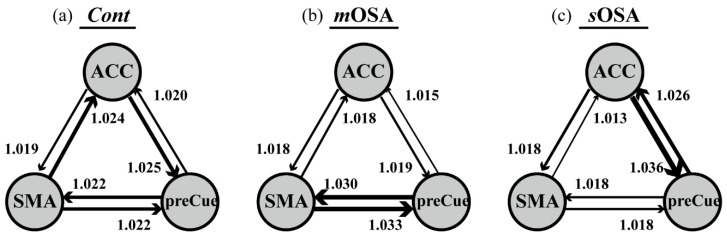
Interactive relationship among the ACC, the precuneus, and the SMA for each group. The thickness of the line denotes the influence between two regions, and the direction of the arrow shows the affective direction from a region to the end of the arrowed region with the impact value. In the *Cont* group (**a**), the three regions are connected by balanced weights. In patients with *m*OSA (**b**), the connectivity to the other two regions for the ACC was reduced, and the network balance is tilted toward the connections between the SMA and the precuneus. In patients with *s*OSA (**c**), the ACC showed increased interactive connectivity to the precuneus, and thus the connection weight is tilted toward the connection of the ACC and the precuneus. This indicates that the pattern of functional connectivity alternates with the severity of OSA, and reflect the vital role of the ACC in the action-monitoring function. Abbreviation: *Cont*, Control group; *m*OSA, moderate OSA group; *s*OSA, severe OSA group; ACC, anterior cingulate cortex; SMA, supplementary motor area; preCue, precuneus; OSA, obstructive sleep apnea.

Figure 4 shows the interregional relationship among the three regions for each group. As shown in the figure, the regions are connected by balanced weights in the *Cont* group (the overall interactive contribution was between 1.020 and 1.025). However, the connectivity to the other two regions for the ACC was reduced in patients with *m*OSA (the interactive contribution was <1.020), indicating that the network balance is tilted toward the connections between the SMA and the precuneus (the overall interactive contribution was >1.030). In patients with *s*OSA, the ACC showed increased interactive connectivity to the precuneus, and thus the connection weight is tilted toward the connection of the ACC and the precuneus (the overall interactive contribution was >1.025), whereas the other interactive contributions between two regions were <1.020. This indicates that the pattern of functional connectivity alternates with the severity of OSA, and the connectivity alteration changes from balanced connection among the regions to regional domination along with the severity of OSA pathology, especially reflecting the role of the ACC in the action-monitoring function.

### 3.3. The Relationship between Functional Performance and PSG Parameters

**Figure 5:**
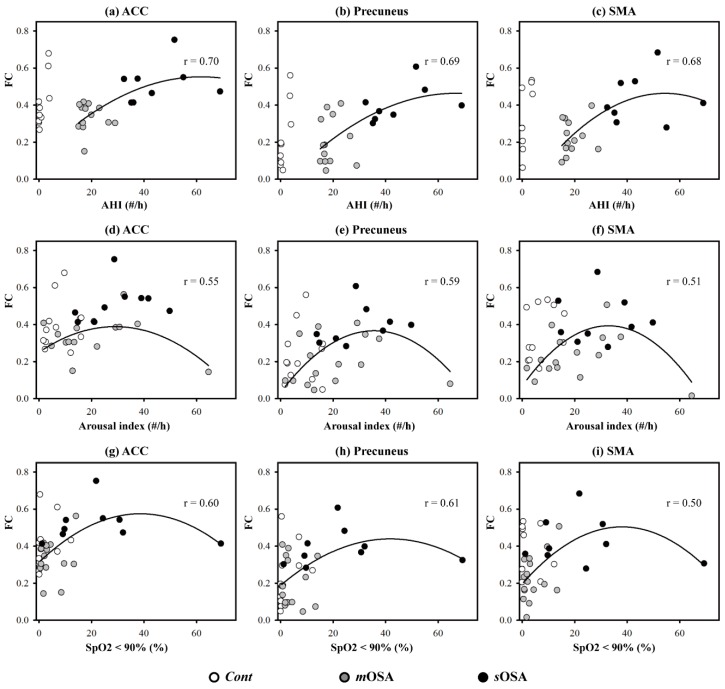
Scattering plot of PSG parameters and functional connectivity. Three regions of interest—the ACC (**a**,**d**,**g**), the precuneus (**b**,**e**,**h**), and the SMA (**c**,**f**,**i**), are demonstrated in each column, and three PSG parameters, including the AHI (**a**–**c**), the arousal index (**d**–**f**), and SpO_2_ (<90%) (**g**–**i**), are presented in each row. Curve fitting was performed with quadratic regression across patients. The fitness value of all the curves was expressed with the correlation intensity. Functional connectivity and PSG parameters exhibited good fitness with a quadratic regression across patients, particularly for the parameter AHI. The tendency of the distribution implied that functional connectivity would become stronger from *m*OSA to *s*OSA, and that it would decline when the OSA symptom worsened. Abbreviation: AHI, apnea-hypopnea index; FC, functional connectivity; *Cont*, Control group; *m*OSA, moderate OSA group; *s*OSA, severe OSA group; ACC, anterior cingulate cortex; SMA, supplementary motor area; OSA, obstructive sleep apnea; PSG, polysomnography.

Figure 5 depicts the scattering plots of functional connectivity on the three regions and the PSG parameters in terms of the AHI, the arousal index, and SpO_2_ (value < 90%) across the three study groups. Functional connectivity and PSG parameters exhibited good fitness with a quadratic regression across patients, showing a fitness value implying the interpretation coverage of data variance. The fitness value of all the curves was >0.5, whereas the fitness value approached 0.7 for the parameter AHI. The tendency of the distribution implied that functional connectivity would become stronger from *m*OSA to *s*OSA, and that it would decline when the OSA symptom worsened. This result supported our hypothesis that functional compensation would occur in *m*OSA and *s*OSA.

## 4. Discussion

To the best of our knowledge, this case–control study is the first investigation to examine the action-monitoring function in patients with OSA using comprehensive behavioral, neurophysiological, and functional image evaluation. The primary findings are consistent with our hypothesis that patients with varying severities of OSA exhibit varying alterations in behavioral and functional performance underlying the action-monitoring function. In our study, patients with *m*OSA exhibited reduced amplitudes of both ERN and Pe accompanied by impaired performance in the modified Flanker task, which indicated action-monitoring dysfunction in experimental sleep research [19,21,37]. Consistently, the resting-state fMRI data also provided evidence of action-monitoring dysfunction in patients with *m*OSA on the ACC functional network in our study. Using fMRI, we demonstrated that *m*OSA is associated with decreased cerebral activation in multiple ACC functional-connected areas, compared with that in the *Cont* group, suggesting that a compromised cerebral function in response to cognitive challenges underlies the action-monitoring dysfunction in patients with *m*OSA.

Among factors responsible for action-monitoring dysfunction, sleep fragmentation and intermittent hypoxia may contribute to the dysfunction observed in patients with OSA. Sleep fragmentation disrupts sleep architecture and reduces the efficacy of restorative processes in sleep [2]. Intermittent hypoxia increases oxidative stress and results in neuroinflammation in the frontal lobe [38]. It has been reported that the combined drawbacks of sleep fragmentation and intermittent hypoxia result in decreased regional cerebral blood flow in the bilateral cingulate gyrus [39]. Furthermore, the duration in hypoxia and the frequency of sleep fragmentation could contribute to decreased ACC activations in patients with OSA [22,23]. Based on the abovementioned concept, cerebral gray matter deficits, impaired white matter integrity, and functional disconnection in the ACC were apparently detected in patients with OSA [40,41,42]. Overall, the pathophysiological impact of intermittent hypoxia and sleep fragmentation induces ACC damage, thereby contributing to the changes in ERN and Pe, altered ACC network, and finally leading directly to action-monitoring dysfunction in the moderate stage of OSA.

Another innovative finding observed in this study is that patients with *s*OSA exhibited increased amplitudes of ERN and Pe and increased cerebral activation in multiple ACC functional-connected areas in the resting-state fMRI compared to those in patients with *m*OSA. Increased neural activities and functional connectivity in the ACC in patients with *s*OSA can be interpreted either as compensatory neural recruitment to maintain action-monitoring performance or as nonselective recruitment interfering with action-monitoring performance [43,44]. To address this issue, the action-monitoring performances of patients with *m*OSA and those with *s*OSA were examined and compared. A trend of deterioration in performances was observed in the modified Flanker task between patients with *m*OSA and those with *s*OSA, as shown in Table 1. However, the post hoc analysis demonstrated no significant difference in the error response rate and the posterror correction rate in both the congruent and incongruent modified Flanker tasks between these two groups, which supports the finding that increased activation and functional connectivity in the ACC in patients with *s*OSA may represent effective recruitment of neurons beyond those used by patients with *m*OSA, to compensate the trend of deterioration in the action-monitoring function. Therefore, we suggest that increased activation and functional connectivity in the ACC-associated cerebral regions to maintain action-monitoring performance in patients with *s*OSA represents a compensatory response.

The functional recruitment in patients with moderately severe OSA is supported by other cognitive evidence such as default mode networking, verbal learning skill, and working memory capability [45,46,47]. Patients with OSA (mean AHI = 54.7) have been shown to demonstrate increased functional connectivity of the default mode in the posterior cingulate cortex, which might reflect neural compensation for cognitive damage [45]. Furthermore, patients with OSA (mean AHI = 35.1) exhibited intact performance in verbal learning, accompanied by increased cerebral activations in the associated cerebral regions compared with those in healthy subjects [46]. In an evaluation of working memory in another study, the results showed that with an increase in severity (mean AHI = 40.1), there was an increase in functional activations in the right parietal lobe, which may potentially reflect compensatory neural responses in patients with moderately severe OSA [47]. To summarize, the findings in the literature together with our findings lead us to interpret that these increased cerebral activations in functional connectivity serve to compensate for natural neuronal activity to maintain the action-monitoring function in patients with moderately severe OSA.

In addition to the changes in functional performance in specific regions, this study demonstrated the relationships among action-monitoring-related regions in the functional network. It has been reported that patients with OSA suffer from impaired network organization, resulting in dysfunctions of autonomic, affective, executive, sensorimotor, and cognitive functions [48]. However, the functional network underlying the action-monitoring function is not well known. We selected the ACC, the precuneus, and the SMA for analyzing the functional connectivity because their functional roles have been specified in the essence of the action-monitoring function. SMA plays the leading role in early response-conflict monitoring by censoring the responses to correct or erroneous behavioral actions [49]. Driven along the path of signal migration from the SMA, the ACC is closely involved in error processing [31]. Furthermore, it has been reported that the activation response of the precuneus is associated with the adaptation or adjustment of successive actions in error processing, and this is required to shift attention to prevent successive error responses [32,33,50]. In summary, a highly functional link exists between the SMA, the ACC, and the precuneus in the processing of action-monitoring.

Our findings emphasized the role of the ACC in the action-monitoring function in the progression of OSA pathology; that is, the connection of the ACC to the SMA and the precuneus declines in the stage of moderate OSA, but it reflects reinforced connection to the precuneus in the stage of severe OSA (shown in Figure 4). Our study also demonstrated altered activation in functional connectivity among the SMA, the ACC, and the precuneus based on the severity of OSA. In patients with *m*OSA, the ACC had the most significantly reduced functional connections with the other two regions. In contrast, in patients with *s*OSA, the connections between the ACC and the precuneus were re-enhanced most significantly. Based on the finding of deactivation and disrupted connections of the ACC in patients with *m*OSA and the regained connections of the ACC in patients with *s*OSA, it could be inferred that the ACC plays a vital role in terms of action-monitoring dysfunction, and that it is adaptable in the pathological progression of OSA. Furthermore, we have shown that the phenomenon of functional compensation might drop out as the AHI value exceeds 60 based on our regression model of function connectivity along with AHI values (shown in Figure 5).

To the best of our knowledge, our study might be the first to examine the error-related ERPs in patients with OSA. Moreover, ACC networking associated with the action-monitoring function has never been reported in patients with moderate to moderately severe OSA. Our study results presented the possible occurrence of compensatory neural recruitment to compensate for the action-monitoring dysfunction in patients with moderately severe OSA based on comprehensive behavioral, electrophysiological, and functional image evaluation and supported an additional insight into neuronal plasticity for patients with OSA.

However, we should acknowledge the limitations of this study. First, although our data have passed the homogeneity of variance test on baseline information among the study groups using Levene’s test, it is also important to consider the issue of performance validity due to the small sample size in future work. Furthermore, there was a substantial female predominance in the healthy control group compared with the OSA groups. The impact of sex on cognitive impairment in OSA has not been determined before. Due to the small sample size, it was not possible to perform subgroup analysis stratified by sex in this study. Second, our study was aimed at investigating the action-monitoring function. To avoid the possible interference of global cognitive function in the action-monitoring function, we excluded subjects with CASI score below the cutoff value. This inclusion criterion limited our participants to moderately severe OSA, because very severe OSA could contribute to obvious multiple cognitive impairment. Third, we did not include patients with mild OSA in this study. Previous studies have reported that patients with mild OSA might not show appreciable deficits in overall cognition and neuropsychological tests [51,52]. Moreover, due to the hospital-based study design, it was difficult to enroll adequate number of patients with mild OSA. The symptoms of mild OSA might be too subtle to make these patients seek medical advice. Therefore, we were not able to determine a dose-dependent response for action-monitoring dysfunction over the complete range of severity of OSA. Further longitudinal studies addressing these issues could be considered to identify and confirm the changes in cerebral responses for the action-monitoring function in patients over the complete range of severity of OSA.

## 5. Conclusions

Using the modified Flanker task, error-related ERPs, and fMRI, this study has demonstrated that OSA impaired the action-monitoring function, but compensatory neural recruitment and cerebral activation were possible in patients with moderately severe OSA. Function connectivity and network organization in the ACC play an important role in governing the changes in action-monitoring-related cerebral responses, which is adaptable along with the severity of OSA. This study emphasizes the importance of early treatment for OSA, when cerebral plasticity is preserved, to decrease the burden of permanent cognitive impairment.

## Figures and Tables

**Table 1:** Demographic information, polysomnography parameters, and performance on the modified Flanker task in the three study groups.

	*Cont* (*n* = 14)	*m*OSA (*n* = 17)	*s*OSA (*n* = 10)	*p* value	$\eta_{p}^{2}$
Gender (males, %) ^†^	6 (42.9)	13 (76.5)	7 (70.0)	0.136	-
Age (year)	42.5 (2.4)	49.2 (1.8)	50.7 (3.7)	0.183	0.095
Education (years)	14.5 (0.7)	13.6 (0.6)	12.9 (0.8)	0.640	0.026
CASI (total scores)	96.3 (1.2)	93.6 (1.0)	93.7 (1.4)	0.183	0.095
AHI (#/h)	1.2 (0.4)	19.8 (1.1)	45.6 (3.8)	<0.001 **	0.878
Arousal index (#/h)	8.2 (1.4)	20.6 (3.7)	29.6 (3.7)	<0.001 **	0.347
SpO_2_ < 90% (%)	4.1 (2.0)	4.7 (1.1)	21.6 (6.3)	0.001 *	0.326
ESS	7 (0.7)	10.4 (1.1)	9.1 (1.0)	0.036 *	0.183
Error response rate (%)					
Congruent	1.6 (0.9)	13.9 (6.3)	18.7 (8.3)	0.122	0.105
Incongruent	4.9 (1.0)	21.7 (7.7)	25.2 (8.8)	0.091	0.118
pError correction rate (%)					
Congruent	86.3 (11.8)	70.4 (9.1)	53.2 (13.4)	0.195	0.110
Incongruent	89.8 (6.6)	74.2 (6.0)	64.1 (7.9)	0.048 *	0.148

^†^ Chi-squared test; the other items: one-way ANOVA. * *p* < 0.05; ** *p* < 0.001. Note: All values denote mean (±standard error). Abbreviations: OSA, obstructive sleep apnea; *Cont*, Control group; *m*OSA, moderate OSA group; *s*OSA, severe OSA group; CASI, Cognitive Abilities Screening Instrument; AHI, apnea–hypopnea index; SpO_2_, blood oxygen saturation; ESS, Epworth Sleepiness Scale; pError, posterror.

## Supplementary Materials

The following are available online at https://www.mdpi.com/2077-0383/8/7/1077/s1, Figure S1: Activation information within clusters.
