# Supplementary material for: Compensatory Neural Recruitment for Error-Related Cerebral Activity in Patients with Moderate-To-Severe Obstructive Sleep Apnea
Source: J Clin Med. 2019 Jul 22;8(7):1077. doi: 10.3390/jcm8071077 (PMC6678110; doi:10.3390/jcm8071077)
Supplement: Supplementary file 1 [file jcm-08-01077-s001.pdf]

Table S1. Activation information within clusters. Between-group differences are the results of *t* tests ($p < 0.05$) and additional cluster constraint ($p < 0.05$). The comparisons are as follows: (a) *s*OSA vs. *Cont*; (b) *m*OSA vs. *Cont*; and (c) *s*OSA vs. *m*OSA.

	side	voxels	t-max	x	y	z	side	voxels	t-max	Coordination (x,y,z)		
(a) *s*OSA vs *Cont*												
ACC	L	20	3.77	-9	30	-9	R	21	3.11	3	45	18
MCC	L	39	3.80	-6	-45	33	R	100	3.37	12	-36	33
PCC	L	69	3.92	-9	-48	30	R	40	3.73	3	-57	30
preFrontal C.	L	623	4.92	-9	27	-12	R	436	3.71	42	45	-15
Insula	L	24	3.96	-39	-12	6	R					
preCentral C.	L	145	4.51	-51	12	39	R					
postCentral C.	L	146	3.65	-51	-6	15	R					
Inf. Parietal C.	L	143	3.67	-36	-72	45	R	10	3.09	57	-60	39
Sup. Parietal C.	L	27	5.47	-30	-72	54	R	8	3.12	36	-78	48
Temporal C.	L	47	3.34	-45	-12	0	R	470	4.32	69	-30	-15
Precuneus	L	73	3.44	0	-57	33	R	134	4.51	3	-57	33
Fusiform	L						R	29	4.19	42	-12	-27
Heschl	L	23	3.18	-45	-12	3	R					
Rectus	L	60	5.15	-9	36	-21	R					
Rolandic Oper	L	45	3.69	-51	-6	12	R					
SupraMarginal	L	22	2.45	-48	-27	30	R	9	2.52	57	-48	30
Angular	L	218	3.77	-39	-57	27	R	195	4.20	57	-63	36
Occipital C.	L	70	3.34	-33	-66	39	R	72	3.04	36	-69	24
(b) *m*OSA vs *Cont*												
	side	voxels	t-max	x	y	z	side	voxels	t-max	Coordination (x,y,z)		
MCC	L	12	-2.08	0	-42	51	R	20	-2.08	9	21	39
Insula	L						R	23	-2.10	39	9	-9
Sup. Parietal C.	L	34	-2.07	-18	-54	69	R					
Temporal C.	L	10	-2.08	-33	-36	-15	R	24	-2.10	45	-9	-3
Precuneus	L	60	-2.08	-9	-48	75	R	15	-2.09	15	-48	6
Calcarine	L	99	-2.08	-6	-72	21	R	123	-2.08	9	-69	6
Cuneus	L	21	-2.13	-9	-75	21	R	9	-2.10	6	-84	15
Fusiform	L	10	-2.08	-33	-24	-18	R					
Lingual	L						R	15	-2.08	6	-72	0
Occipital C.	L	30	-2.09	-21	-75	27	R					
(b) *s*OSA vs *m*OSA												
	side	voxels	t-max	x	y	z	side	voxels	t-max	Coordination (x,y,z)		
Caudate	L	12	4.49	-6	9	-12	R	23	3.65	12	12	-6
ACC	L	71	4.19	-9	39	-6	R	75	3.64	9	39	0
MCC	L	103	3.64	-3	-42	51	R	191	3.86	6	-9	33
PCC	L	59	5.40	-6	-45	21	R	62	3.91	3	-45	21
Olfactory	L	20	3.91	0	9	-9	R					
preFrontal C.	L	1086	4.71	-42	48	9	R	1128	4.54	36	9	51
SMA	L	66	3.35	-6	-15	57	R	109	3.34	3	-24	57
Insula	L	47	3.81	-33	-21	9	R	47	3.16	42	-12	6
preCentral C.	L	277	4.25	-51	0	51	R					
postCentral C.	L	221	4.71	-45	-9	45	R	10	2.61	69	-9	18
Hippo.	L	72	3.89	-27	-21	-18	R	47	3.61	39	-12	-24
ParaHippo.	L	40	4.58	-18	-27	-18	R	78	3.32	21	-36	-12
Inf. Parietal C.	L	183	4.08	-45	-51	39	R	11	2.52	48	-48	42
Sup. Parietal C.	L	43	2.83	-33	-60	60	R	6	3.61	36	-78	48
Temporal C.	L	409	4.20	-63	-21	-15	R	897	5.91	69	-30	-12
Precuneus	L	134	3.42	0	-75	42	R	242	4.39	9	-54	36
Fusiform	L	36	4.53	-36	-12	-27	R	78	4.45	39	-12	-27
Heschl	L	50	4.32	-36	-21	9	R	41	3.18	39	-24	12
Rectus	L	77	5.57	-9	54	-15	R	41	4.52	9	54	-18
Rolandic Oper	L	67	3.22	-48	-12	12	R	42	2.84	42	-33	21
SupraMarginal	L	40	3.15	-48	-27	33	R					
Angular	L	245	4.19	-39	-60	24	R	312	5.14	45	-66	45
Cuneus	L	41	3.30	-15	-66	24	R	59	3.27	12	-78	39
Lingual	L	10	2.78	-12	-33	-3	R	24	2.97	18	-36	-12
Calcarine	L						R	146	3.68	12	-75	12
Occipital C.	L	254	4.81	-33	-72	24	R	482	4.14	33	-75	27
